# Pre- and postoperative need for pituitary hormone replacement in non-adenomatous sellar and parasellar lesions: importance of the sellar encroachment score

**DOI:** 10.1007/s00701-020-04440-4

**Published:** 2020-06-06

**Authors:** Mueez Waqar, Shiva Rampersad, David Bennett, Tara Kearney, Kanna K. Gnanalingham

**Affiliations:** 1 Department of Neurosurgery, Manchester Centre for Clinical Neurosciences (MCCN), Salford Royal Foundation Trust (SRFT), Stott Lane, Salford, M6 8HD UK; 2 Manchester Academic Health Science Centre, The University of Manchester, Manchester, UK; 3 Endocrinology, Salford Royal NHS Foundation Trust, Stott Lane, Salford, UK

**Keywords:** Non-adenomatous, Pituitary, Hormones, Sellar region, Encroachment score

## Abstract

**Background:**

Pre-/postoperative pituitary endocrine deficiencies in patients with sellar/parasellar non-adenomatous lesions are poorly described and studies have not considered the effect of sellar invasion on endocrine outcome. The aim of this study was to relate the need for pituitary hormone replacement pre-/postoperatively, with sellar invasion, in non-adenomatous sellar/parasellar lesions.

**Methods:**

Single-centre review of adults with histologically confirmed non-adenomatous sellar/parasellar lesion and follow-up ≥ 3 months or until postop radiotherapy. Pituitary dysfunction was defined by hormone replacement. The sellar encroachment score (0–6) was calculated as the sum of the thirds of radiological encroachment into the sellar region in the coronal and sagittal planes. Multivariate analysis with binary logistic regression was used to determine factors associated with pituitary hormone replacement.

**Results:**

One hundred and seventeen patients were included with a median age of 49 years (range 16–84 years) and median follow-up of 13 months. Surgery was trans-sphenoidal (53%), trans-cranial (36%) or a combination (11%). The commonest histology types were meningioma (*n* = 33, 28%) and craniopharyngioma (*n* = 20, 17%). The median sellar encroachment score was 6 (range 0–6). Most (*n* = 86, 74%) did not require pituitary hormone replacement preoperatively. The need for pituitary hormones increased after surgery in 41 (35%) patients. In multivariate analysis, the sellar encroachment score was the only factor predictive of pre- (OR = 2.6, 95% CI = 1.2–5.5; *p* = 0.01) and postoperative risk of new pituitary hormone replacement (OR = 4.1, 95% CI = 1.7–10.1, *p* = 0.002).

**Conclusion:**

A significant proportion of these patients present with need for pituitary hormone replacement that may worsen postoperatively. The degree of sellar encroachment is predictive of pituitary hormone replacement status pre-/postoperatively.

## Introduction

Patients with sellar region lesions can present with hypopituitarism. However, endocrine outcomes are best described for the subgroup of pituitary adenomas, which constitute over 90% of the lesions in this area [13]. The remaining 10% of patients harbour other non-adenomatous pathology, which can range from other tumour subtypes (e.g. meningiomas) to cysts, vascular malformations, inflammatory processes and infection [13]. There is a sparsity of literature describing outcomes in this group, especially those related to pituitary endocrine function. This is important as hypopituitarism has previously been identified as an independent predictor of mortality [12]. Outcome data can help guide clinicians as to which patients at presentation need screening for pituitary dysfunction at presentation, and communicate the risk of hypopituitarism with surgery, particularly for those patients with sellar/parasellar pathology types at risk of requiring postop hormone replacement.

Studies to data describing outcomes in non-adenomatous sellar/parasellar lesions have reported conflicting findings [2, 3, 8]. This is for a variety of reasons. Some studies have restricted their inclusion criteria and reported on the commonest histology types encountered in this region, whilst others have focussed on the rarest [3, 8]. The majority have also not controlled for the effects of radiotherapy, which can cause irreversible and progressive pituitary dysfunction [2]. Other important considerations include the lesion size, the extent of resection and potentially the degree of encroachment of the lesion into the pituitary fossa.

The aim of the present study was to present pituitary endocrine outcomes as assessed by need for pituitary hormone replacement in a large cohort of non-adenomatous lesions of the sellar/parasellar region. We accounted for postoperative radiotherapy use and also considered the degree of sellar encroachment by developing a novel scoring system.

## Methods

This single-centre study was registered locally and approved by the institutional review board. Ethical approval, patient consent and funding were not required for this study design.

### Data collection

Adult (≥ 16 years) patients with histologically confirmed non-adenomatous sellar and parasellar lesions were identified from a prospective pituitary database operated on by a single surgeon (2006–2018). Follow-up was defined as the time interval between surgery and the last clinic appointment, or in the case of patients undergoing postoperative radiotherapy, the last clinic appointment before delivery of radiotherapy.

Data was collected on patient demographics, MRI characteristics, histology, treatment and pituitary hormone status preoperatively and at last follow-up. Histology types were grouped as in previous studies [2, 3, 8]. All patients had pre- and postoperative baseline pituitary hormone profile assessed by the endocrine team. Postoperative tests included dynamic assessment (i.e. glucagon stress test) of pituitary hormone secretion. Pituitary hormone status was recorded prior to commencement of postoperative radiotherapy where applicable. Pituitary dysfunction was defined in terms of replacement hormone required for each key pituitary hormone axis—glucocorticoids (ACTH), thyroxine (TSH), growth hormone (GH) and desmopressin (ADH). With respect to gonadotrophs and sex hormones, we took note of replacement with testosterone and oestrogen/progesterone.

### Imaging review

Preoperative MRI scans were reviewed by SR and DB to determine lesion volume as described previously (4/3 × π × radius^3^) [14].

**Fig. 1:**
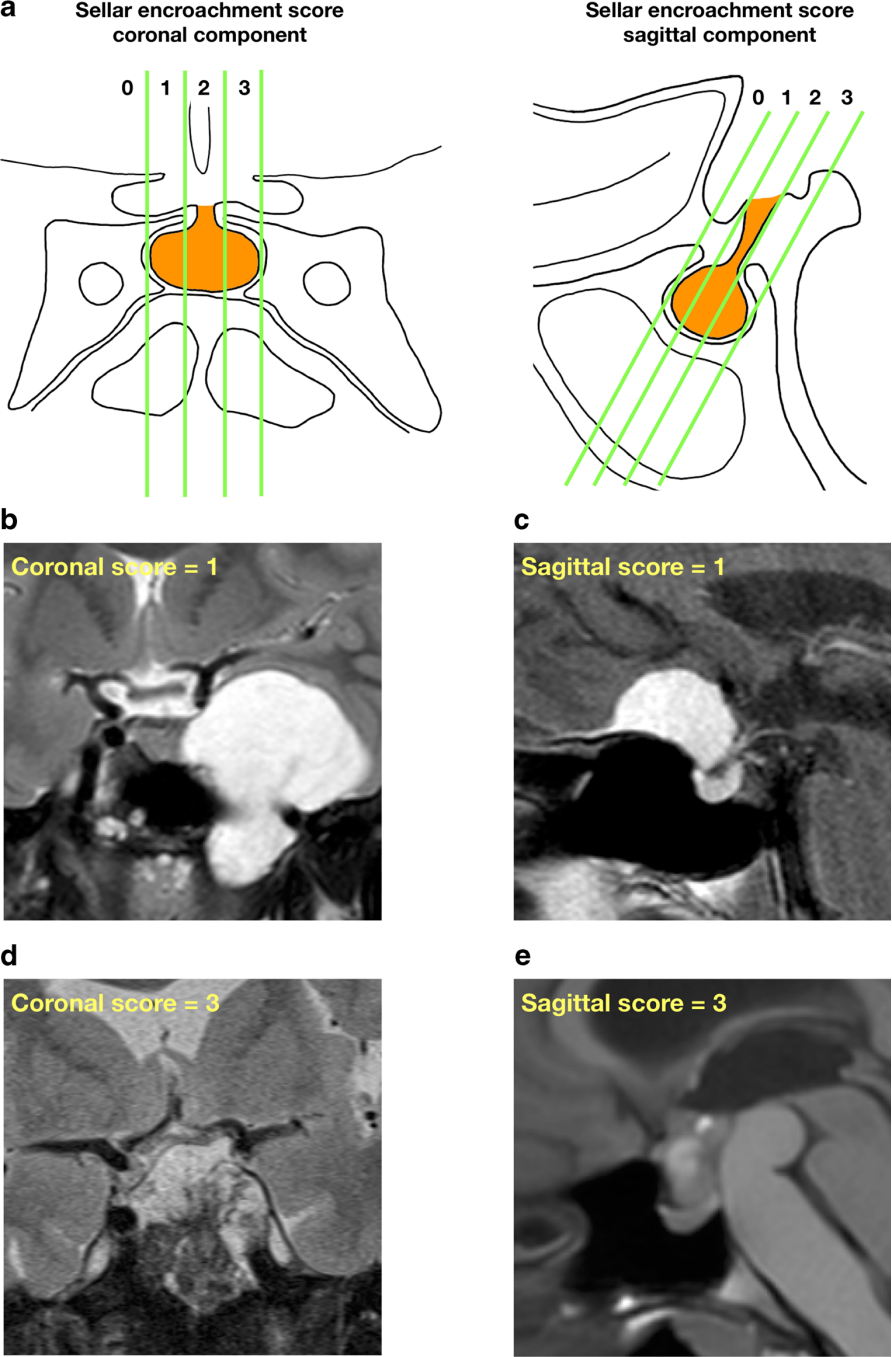
Sellar encroachment score. **a** The total score is the sum of the thirds of sellar encroachment in the coronal (left panel) and sagittal (right panel) planes. For each plane, imaginary lines parallel to the pituitary stalk dividing the sellar into thirds are considered. Sellar encroachment can be from any direction, but in these figures it is drawn in an advancing manner from the left to right and anterior to posterior directions. A score of 0 indicates no encroachment into the sellar floor. **b** A 27-year-old female with an incidental left cavernous lesion underwent a trans-sphenoidal biopsy and later a craniotomy for a left temporal fossa chondrosarcoma. She did not require any pituitary hormone replacement pre- or postoperatively. **c** A 77-year-old male with left-sided visual field deficits underwent trans-nasal resection of a tuberculum sellar meningioma (WHO grade 1). Pre- and postoperatively the patient was on thyroxine for primary hypothyroidism. **d** A 70-year-old male presented with diplopia and left-sided ptosis and underwent trans-sphenoidal resection of a large sellar region chordoma. Preoperatively the patient required hydrocortisone and thyroxine with additional need for testosterone postoperatively. **e** A 20-year-old female presented with headaches and blurred vision. A suprasellar lesion with associated prepontine cyst was debulked via a craniotomy with insertion of Ommaya reservoir into the prepontine cyst. Histology was consistent with craniopharyngioma. Preoperatively, the patient did not require any hormone replacement. Postoperatively, she required thyroxine

The degree of encroachment into the pituitary fossa in the sagittal and coronal planes was assessed using the sellar encroachment score (Fig. 1a). The sellar encroachment score (0–6) represented the sum of the number of thirds of sellar encroachment in the coronal (Fig. 1a left panel) and sagittal planes (Fig. 1a right panel). Sellar encroachment can be from any direction, but in this figure it is drawn in an advancing manner from right to left and anterior to posterior directions. A score of 0 indicates no encroachment into the sellar floor.

Serial postoperative MRI scans were also reviewed to determine the extent of resection: biopsy (≤ 10%), subtotal (10–90%) and near total (i.e. > 90%; no obvious remnant evident).

The sellar encroachment score (0–6) represented the sum of the number of thirds of sellar encroachment in the coronal (Fig. 1a left panel) and sagittal planes (Fig. 1a right panel). Sellar encroachment can be from any direction and a score of 0 indicates no encroachment into the sellar floor.

Serial postoperative MRI scans were also reviewed to determine the extent of resection: biopsy (≤ 10%), subtotal (10–90%) and near total (i.e. > 90%; no obvious remnant evident).

### Statistics

Statistical analysis was performed using SPSS version 22 (SPSS Inc., Chicago, IL, USA). Categorical variables were compared using tests of proportions (Fisher’s exact, chi-squared). Inter-rater reliability was performed using the intraclass correlation coefficient indicating a good level of agreement (ICC = 0.74). Multivariate analysis using binary logistic regression was performed to determine factors associated with need for preoperative pituitary hormone replacement and development of additional pituitary hormones postoperatively. Variables were included in the multivariate model where *p* ≤ 0.1 at the univariate level. In multivariate analysis, each variable was given with an odds ratio (OR) and 95% confidence interval (CI).

## Results

One hundred nineteen patients were originally identified from our database and 2 were excluded due to loss to follow-up. One hundred seventeen patients were therefore included in this study. The median age was 49 years (range 16–84 years; mean age 50 years). There was a slight female excess (M:F, 51:66). The median follow-up was 13 months (range 1–117 months; mean 24 months) and the endocrine status was noted before radiotherapy if applicable. Baseline demographic and treatment characteristics are shown in Table 1.

**Table 1:** Patient baseline demographic and treatment characteristics

*N*	117
Age—median (range)	49 (16–84)
Gender	
Male	51 (44%)
Female	66 (56%)
Surgical approach	
Trans-sphenoidal	62 (53%)
Cranial or endoscopic	42 (36%)
Combination	13 (11%)
Number of operations	
1	92 (79%)
2	21 (18%)
3	2 (2%)
4	2 (2%)
Extent of surgical resection	
Biopsy	10 (9%)
Subtotal resection	66 (56%)
Gross total resection	41 (35%)
Radiotherapy	
Yes	37 (32%)
No	80 (68%)
Histology	
Meningioma	33 (28%)
Craniopharyngioma	20 (17%)
Rathke’s cyst	9 (8%)
Inflammatory/infective lesion	10 (9%)
Other neoplasm (e.g. chordoma, metastasis, glioma)	25 (21%)
Cystic lesions (dermoid, epidermoid, pituitary cyst, meningoencephalocele)	12 (10%)
Other (e.g. cavernoma)/non-diagnostic	8 (7%)
Sellar encroachment score	
0	17 (15%)
1	1 (1%)
2	5 (4%)
3	1 (1%)
4	12 (10%)
5	15 (13%)
6	66 (56%)

The surgical approach was either trans-sphenoidal (*N* = 62, 53%), craniotomy (*N* = 42, 36%) or a combination of these (*N* = 13, 11%). Radiotherapy was administered postoperatively in 37 (32%) patients. The median number of operations before radiotherapy was 1 (range 1–4) in 92/117 patients (79%). Based on the serial postoperative MRI scans, the extent of surgical resection was considered to be a biopsy (*N* = 10, 9%), subtotal resection (*N* = 66, 56%) or near total resection (*N* = 41, 35%).

The commonest histology types were meningioma (*N* = 33, 28%), craniopharyngioma (*N* = 20, 17%) and Rathke’s cyst (*N* = 9, 8%). Other neoplasms encountered included chordoma (*N* = 6, 5%), chondrosarcoma (*N* = 7, 6%), metastases (*N* = 2, 2%), schwannoma (*N* = 1, 1%), astrocytoma (*N* = 2, 2%) and myeloma (*N* = 1, 1%). Vascular lesions were rare, with two cases of cavernoma (2%) and one haematoma (1%). Cystic lesions included dermoid cysts (*N* = 1, 1%), epidermoid cysts (*N* = 2, 2%), pituitary cysts (*N* = 1, 1%) and meningoencephalocele (*N* = 5, 4%). No histological diagnosis was reached in four cases (4%).

The median lesion volume was 4.19 cm^3^ (range 0.07–64.2 cm^3^, mean 7.33 cm^3^) and the median sellar encroachment score was 6 (range 0–6, mean 5) (Table 1).

Endocrine outcomes pre- and postoperatively are shown in Table 2. Preoperatively, most patients (*N* = 86, 74%) did not require pituitary hormone replacements. No patient had panhypopituitarism preoperatively. Preoperatively in patients needing at least one pituitary hormone replacement (*N* = 31, 26%), the most common pituitary hormone replacements were thyroxine (*N* = 23) and glucocorticoids (*N* = 20).

**Table 2:** Pre- and postoperative pituitary hormone replacements (*N* = 117). *NA*, not applicable

	Preoperative (*N* = 117)	Postoperative (*N* = 117)
Number of pituitary hormone replacements		
0	86 (74%)	59 (50%)
1	17 (15%)	26 (22%)
2	7 (6%)	11 (9%)
3	6 (5%)	9 (8%)
4	1 (1%)	11 (9%)
5	0 (0%)	1 (1%)
Change in number of pituitary hormone replacements postsurgery		
Unchanged	NA	72 (62%)
Reduced	NA	4 (3%)
Increased	NA	41 (35%)
Types of pituitary hormone replacements required		
*N*, ≥ 1 axis pituitary hormones	31	58
Glucocorticoids	20/31 (65%)	39/58 (67%)
Thyroxine	23/31 (74%)	42/58 (72%)
Growth hormone	1/31 (3%)	9/58 (16%)
Sex hormones	4/31 (13%)	22/58 (38%)
Desmopressin	5/31 (16%)	13/58 (22%)
New pituitary hormone replacements required postoperatively		
*N*	NA	41
Glucocorticoids	NA	23/41 (56%)
Thyroxine	NA	19/41 (46%)
Growth hormone	NA	8/41 (20%)
Sex hormones	NA	18/41 (44%)
Desmopressin	NA	8/41 (20%)

Postoperatively, additional new pituitary hormone replacement was required in 41/117 (35%) patients (Table 2). This usually manifested as one additional pituitary hormone (*N* = 24/41, 59%). The commonest new postoperative hormone replacements were glucocorticoids (*N* = 23/41, 56%) or thyroxine (*N* = 19/41, 46%). A minority (*N* = 4/41, 3%) of patients required fewer hormones after surgery. These 4 patients were on glucocorticoids preoperatively that was not required after surgery.

Preoperatively, multivariate analysis was undertaken to determine factors associated with the need for pituitary hormone replacement (Table 3). Only the sellar encroachment score was associated with preoperative pituitary hormone replacements on uni- and multivariate analysis (OR 2.6; 95% CI = 1.2–5.5; *p* = 0.01).

**Table 3:** Multivariate analysis of factors associated with need for preoperative pituitary hormone replacements (*N* = 31/117; 26%). Binary logistic regression revealed the sellar encroachment score to be the only factor predictive of this outcome

Factor	Preop pituitary hormone replacement (*N*, %)	Univariate analysis	Multivariate analysis
Age			
≤ 49	14/59 (24%)	Fisher’s exact, *p* = 0.54	Not included
> 49	17/58 (29%)		
Gender			
Male	18/51 (35%)	Fisher’s exact, *p* = 0.09	Not included
Female	13/66 (20%)		
Histology			
Meningioma	7/33 (21%)	Chi-squared = 2.94, *p* = 0.4	OR = 1.18, 95% CI = 0.83–1.67, *p* = 0.37
Craniopharyngioma	7/20 (35%)		
Rathke’s	4/9 (44%)		
Other	13/55 (24%)		
Volume			
≤ 4.19 mm^3^	15/60 (25%)	Fisher’s exact, *p* = 0.8	OR = 1.09, 95% CI = 0.46–2.61, *p* = 0.84
> 4.19 mm^3^	16/57 (28%)		
Sellar encroachment score			
0–2	2/23 (9%)	Chi-squared = 6.5, *p* = 0.039	OR = 2.57, 95% CI = 1.21–5.46, *p* = 0.01
3–4	2/13 (15%)		
5–6	27/81 (33%)		

Postoperatively, multivariate analysis was undertaken to determine factors associated with the need for additional new pituitary hormone replacements (Table 4). On univariate analysis, the extent of surgical resection (*p* = 0.019), histology (*p* < 0.001) and sellar encroachment score (*p* < 0.001) were associated with new postoperative pituitary hormone replacements. With respect to histology, craniopharyngiomas had higher (15/20, 75%) rates of requiring new postoperative pituitary hormone replacements compared with meningiomas 12/33 (36%) and Rathke’s cysts 3/9 (33%). In multivariate analysis, only the sellar encroachment score remained significant on multivariate analysis (OR 4.1; 95% CI = 1.7–10.1; *p* < 0.002).

**Table 4:** Multivariate analysis of factors associated with need for new pituitary hormone replacements postoperatively (*N* = 41/117; 35%). Binary logistic regression revealed the sellar encroachment score to be the only factor predictive of this outcome

Factor	New postop pituitary hormone replacements (*N*, %)	Univariate analysis	Multivariate analysis
Age			
≤ 49	21/59 (36%)	Fisher’s exact, *p* > 0.99	Not included
> 49	20/58 (34%)		
Gender			
Male	22/51 (43%)	Fisher’s exact, *p* = 0.12	Not included
Female	19/66 (29%)		
Extent of resection			
Biopsy	1/10 (10%)	Chi-squared = 7.9, *p* = 0.019	OR = 0.55, 95% CI = 0.24–1.22, *p* = 0.14
Subtotal resection	30/66 (45%)		
Gross total resection	10/41 (24%)		
Number of operations pre-radiotherapy			
1	34/92 (37%)	Fisher’s exact, *p* = 0.48	Not included
> 1	7/25 (28%)		
Surgical approach			
Trans-sphenoidal	17/62 (27%)	Chi-squared = 3.6, *p* = 0.17	OR = 0.91, 95% CI = 0.57–1.47, *p* = 0.71
Craniotomy	19/42 (45%)		
Combined	5/13 (39%)		
Histology			
Meningioma	12/33 (36%)	Chi-squared = 19.5, *p* < 0.001	OR = 0.70, 95% CI = 0.47–1.05, *p* = 0.09
Craniopharyngioma	15/20 (75%)		
Rathke’s	3/9 (33%)		
Other	11/55 (20%)		
Volume			
≤ 4.19 mm^3^	20/60 (33%)	Fisher’s exact, *p* = 0.7	Not included
> 4.19 mm^3^	21/57 (37%)		
Sellar encroachment score			
0–2	1/23 (4%)	Chi-squared = 16.7, *p* < 0.001	OR = 4.08, 95% CI = 1.65–10.10, *p* = 0.002
3–4	2/13 (15%)		
5–6	38/81 (47%)		
Preoperative hypopituitarism			
Yes	29/86 (34%)	Fisher’s exact, *p* = 0.66	Not included
No	12/31 (39%)		

Figure 1b–e illustrate typical patient examples with differing levels of encroachment into the sellar region and the respective pituitary hormone outcomes.

**Fig. 2:**
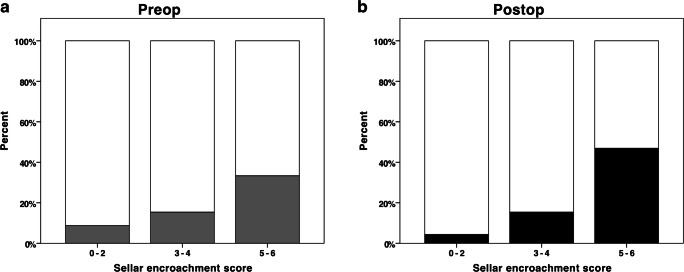
The association between the sellar encroachment score and need for pituitary hormone replacement pre- (**a**) and postoperatively (**b**) (*N* = 117). The grey bar in panel **a** (i.e. preop group) represents the proportion of patients requiring pituitary hormone replacement preoperatively. The black bar in panel **b** (i.e. postop group) represents the proportion of patients with new additional pituitary hormone replacements postoperatively. Pre- (**a**) and postoperatively (**b**) there was increased need for pituitary hormone replacement with increasing sellar encroachment scores

Figure 2 shows a visual representation of need for pituitary hormone replacement pre- and postoperatively in relation to the sellar encroachment score.

## Discussion

Patients with sellar region pathology can present with hypopituitarism which is important to identify given its association with increased morbidity and mortality [12]. In addition, assessing the rate of pituitary dysfunction in such cases is important to guide selection for preoperative pituitary hormone screening and inform patients of the endocrine risks of surgery.

In this single-centre study, we described endocrine outcomes via pituitary hormone replacement in adults with non-adenomatous sellar/parasellar lesions undergoing trans-sphenoidal and/or trans-cranial procedures after a median follow-up of 13 months. More than half (56%) of patients had lesions completely encroaching into the sella in the coronal and sagittal imaging planes preoperatively, though almost three-quarters (74%) did not require pituitary hormone replacement preoperatively. Pituitary status was assessed postoperatively at last follow-up and prior to any radiotherapy, where applicable. A wide variety of pathology subtypes were included. About one-third of patients (35%) required new pituitary hormone replacements postoperatively and a minority (3%) had improved pituitary function. Postoperative deterioration in pituitary function usually manifested as one additional hormone requirement. In multivariate analysis, the sellar encroachment score emerged as the only significant factor predictive of the need for pre- and postoperative new pituitary hormone replacements.

The rate of hypopituitarism would be expected to be lower in non-adenomatous lesions compared with adenomas, which intrinsically involve the pituitary gland. This is exemplified in existing literature and in two large series of pituitary adenomas with 444 and 721 patients, hypopituitarism was noted in 70–86% of patients at presentation [4, 6]. This compares with rates of 26–60% in non-adenomatous case series including ours (see Table 5) [3, 5, 8, 11, 13]. Although the rate of hypopituitarism may be lower for non-adenomatous lesions of the sellar/parasellar region, it is still significant and requires pre- and postoperative screening and appropriate patient counselling.

**Table 5:** Studies reporting general rates of pituitary dysfunction in patients with non-adenomatous sella region lesions

Paper	Location	*N*	Average follow-up (range, months)	Average age (years, range)	Average lesion size (range)	Histology subtypes excluded	Radiotherapy effects controlled for	Pituitary dysfunction (*n*, %)	
								Baseline	New postop
Dusick 2008 ^[3]^	CA, USA	81	12 (3–94)	36 (7–78)	NR	Excluded all other except RCC, CP, meningiomas	Yes—excluded	48/81 (59%)	10/77 (13%)
Valassi 2010 ^[13]^	MA, USA	116	42 (0–132)	45 (13–88)	NR	None excluded	No	45/116 (39%)	12/116 (10%)
Koutourousiou 2010 ^[5]^	Athens, Greece	29	NR	48 (15–78)	MLD 21.8 mm (10–50 mm)	Meningiomas excluded	No	13/29 (45%)	NR
Petrakakis 2016^[8]^	Hannover, Germany	20	52.9 (24–86)	44.2 (2–66)	NR	RCC, CP, meningiomas excluded	No	12/20 (60%)	4/20 (20%)
Somma 2017^[11]^	OH, USA	78	NR	50 (NR)	NR	RCC, CP, meningiomas excluded	No	27/78 (35%)	8/78 (10%)
Patrona 2017^[7]^	NY, USA	15	34.4 (15–77)	51.1 (11–87)	Volume 12.74 ml (4.32–35.80 ml)	meningiomas excluded	No	1/15 (7%)*	1/15 (7%)*
Present study	Manchester, UK	117	13 (1–117)	49 (16–84)	Volume 4.19 cm^3^ (0.07–64.15 cm^3^)	None excluded	Yes—excluded	31/117 (26%)	41/117 (35%)

*Study only reported rates of panhypopituitarism. *RCC*, Rathke’s cleft cyst; *CP*, craniopharyngioma; *M*, meningioma; *MLD*, mean largest diameter; *NR*, not recorded

The rate of new pituitary hormone replacement postoperatively (35%) was relatively high in our study compared with others. This could be due to several reasons. Firstly, more than half of our patients had complete encroachment of the sella radiologically preoperatively. Other studies have not reported lesion size or sellar encroachment characteristics and may have included predominantly smaller lesions not fully involving the sella region. Secondly, unlike the present study, several other studies have excluded histology types associated with higher rates of hypopituitarism such as craniopharyngiomas [7, 8, 11]. To our knowledge, only one existing non-adenomatous case series has described endocrine outcomes without excluding any histology subtypes, though it did not report on sellar encroachment or size characteristics [13]. Valassi et al. described endocrine outcomes in a large cohort of non-adenomatous lesions after an average follow-up of 42 months. Their rate of deterioration in postoperative pituitary function was just 10%, though their average follow-up was longer [13]. Pituitary function is known to recover with time after surgery [9].

Almost a fifth of our patients underwent a second surgical procedure and the number of operations was not found to significantly influence endocrine outcome. This is similar to other reports studying pituitary adenomas. For example, Rim et al. described postoperative endocrine status in 29 patients with non-functioning pituitary adenomas undergoing redo surgery. None had worsened endocrine outcomes after 3.6 years average follow-up [10].

We found that the extent of the preoperative sellar encroachment as determined by the sellar encroachment score was predictive of the need for pre- as well as postoperative pituitary hormone replacement. The effect was independent of histology, lesion volume, surgical approach (i.e. cranial versus trans-sphenoidal route) and extent of resection, which did not influence endocrine outcome in multivariate analysis. This is a novel finding. Similar observations have been made in studies investigating pituitary adenomas. Thus, Berkmann et al. described 60 patients with non-functioning pituitary adenomas and found that those with a greater degree of sellar invasion as assessed by the Hardy grade were more likely to develop postoperative hypopituitarism. The effect was independent of tumour volume [1].

We acknowledge a number of limitations in the present study. We have determined pituitary function by the need for hormone replacement rather than objective measurement of pituitary hormone levels or assessment of patient symptomatology. This could have underestimated the true rate of hypopituitarism, though arguably gives a more clinically meaningful result. We also assumed that new postoperative hormone requirements were entirely due to primary hypopituitarism. Other potential causes such as hypothalamic dysfunction, as may arise iatrogenically from surgery, were not considered. Moreover, the temporal pattern of endocrine recovery was not assessed as it is beyond the scope of this study.

## Conclusions

In this study, we describe the risk factors for the need for pre- and postoperative pituitary hormone replacements in one of the largest cohort of patients with non-adenomatous sellar/parasellar lesions in the literature. Around a quarter (26%) required pituitary hormone replacement preoperatively, and 35% required new pituitary hormone replacements postoperatively. We describe a novel score to quantify the degree of sellar encroachment in two imaging planes—the sellar encroachment score, which was the only factor predictive of pituitary endocrine outcome both pre- and postoperatively and before adjuvant radiotherapy. Existing literature on non-adenomatous sellar pathology is limited and our findings require future validation.
